# Combination of structural MRI, functional MRI and brain PET-CT provide more diagnostic and prognostic value in patients of cerebellar ataxia associated with anti-Tr/DNER: a case report

**DOI:** 10.1186/s12883-021-02403-5

**Published:** 2021-09-24

**Authors:** Sisi Shen, Wenyu Liu, Ming Zhou, Ruiyi Yang, Jinmei Li, Dong Zhou

**Affiliations:** 1 Department of Neurology, West China Hospital, Sichuan University, No. 37 GuoXue Alley, 610041 Chengdu, Sichuan China; 2 Department of Radiology, the Third Hospital of Mianyang, Sichuan Mental Health Center, No. 190 East of Jiannan Road, 621000 Mianyang, Sichuan China

## Abstract

**Background:**

Brain magnetic resonance imaging (MRI) rarely reveals structural changes in patients with suspected anti-Tr/DNER encephalitis and thus provides very limited information. Here, we combined structural MRI, functional MRI, and positron emission tomography-computed tomography (PET-CT) findings to characterize this rare disorder in a patient.

**Case presentation:**

A 43-year-old woman presented with progressive cerebellar ataxia, memory impairment, anxiety, and depression. Anti-Tr antibodies were detected in both her serum (1:10) and cerebrospinal fluid (1:10). A diagnosis of anti-Tr-positive autoimmune cerebellar ataxia was established. The patient’s symptoms were worse, but her brain MRI was normal. Meanwhile, voxel-based morphometry analysis showed bilateral reduced cerebellar volume, especially in the posterior lobe and uvula of the cerebellum and the middle of the left temporal lobe compared with 6 sex- and age-matched healthy subjects (6 females, 43 ± 2 years; *p* < 0.05). Using seed-based functional connectivity analysis, decreased connectivity between the posterior cingulate cortex/precuneus and left frontal lobe compared to the control group (*p* < 0.05) was detected. PET-CT revealed bilateral hypometabolism in the cerebellum and relative hypermetabolism in the cerebellar vermis and bilateral frontal lobe, but no malignant changes.

**Conclusions:**

A combination of structural MRI, functional MRI, and brain PET-CT has higher diagnostic and prognostic value than conventional MRI in patients with suspected anti-Tr/DNER encephalitis.

**Supplementary Information:**

The online version contains supplementary material available at 10.1186/s12883-021-02403-5.

## Background

Cerebellar ataxia associated with anti-Tr/DNER (Delta/Notch-like epidermal growth factor-related receptor) autoantibodies is a rare autoimmune disease characterized by progressively acute or sub-acute severe cerebellar ataxia that eventually disables affected patients [1–5]. Progression of this disorder is often irreversible, which is consistent with the total loss of cerebellar Purkinje cells observed at autopsy [4]. A full understanding and early diagnosis of this disease is crucial, as prompt treatment can prevent disability [6]. The major feature of this disorder is severe cerebellar ataxia, which highlights abnormalities of the cerebellum. However, patients often present with other symptoms, such as extensor plantar response [7], retrobulbar optic neuropathy [7], encephalopathy [4], sensory neuropathy and limbic encephalitis [4], which is indicative of the involvement of areas outside the cerebellum. Detecting areas affected in this rare disorder by morphological examination is important, because exploring the associations between affected areas and clinical manifestations may help clarify the pathophysiological mechanisms and predict prognosis of this disease.

However, initial evaluations using conventional brain magnetic resonance imaging (MRI) rarely reveals structural changes [1, 5]. Even when changes are present, they are often subtle or nonspecific, resulting in MRIs providing very limited information. Voxel-based morphometry (VBM), which is an MRI processing technique that can detect regional morphological changes throughout the brain, resting state functional MRI (fMRI), which is an emerging functional imaging technique that analyzes spontaneous fluctuations in the blood oxygen level-dependent (BOLD) signal to assess functional connectivity (FC) of remote brain areas, and positron emission tomography-computed tomography (PET-CT) have been successfully used to detect structural and functional changes in various nervous system diseases. Here, we hypothesize that multimodal imaging analyses may also reveal structural and functional changes in the brains of patients with anti-Tr/DNER cerebellar ataxia, increasing the pathophysiological and prognostic value of these assessments. In this study, we combined MRI with VBM, FC, and PET-CT to assess a patient and characterize this rare disorder.

## Case presentation

**Fig. 1:**
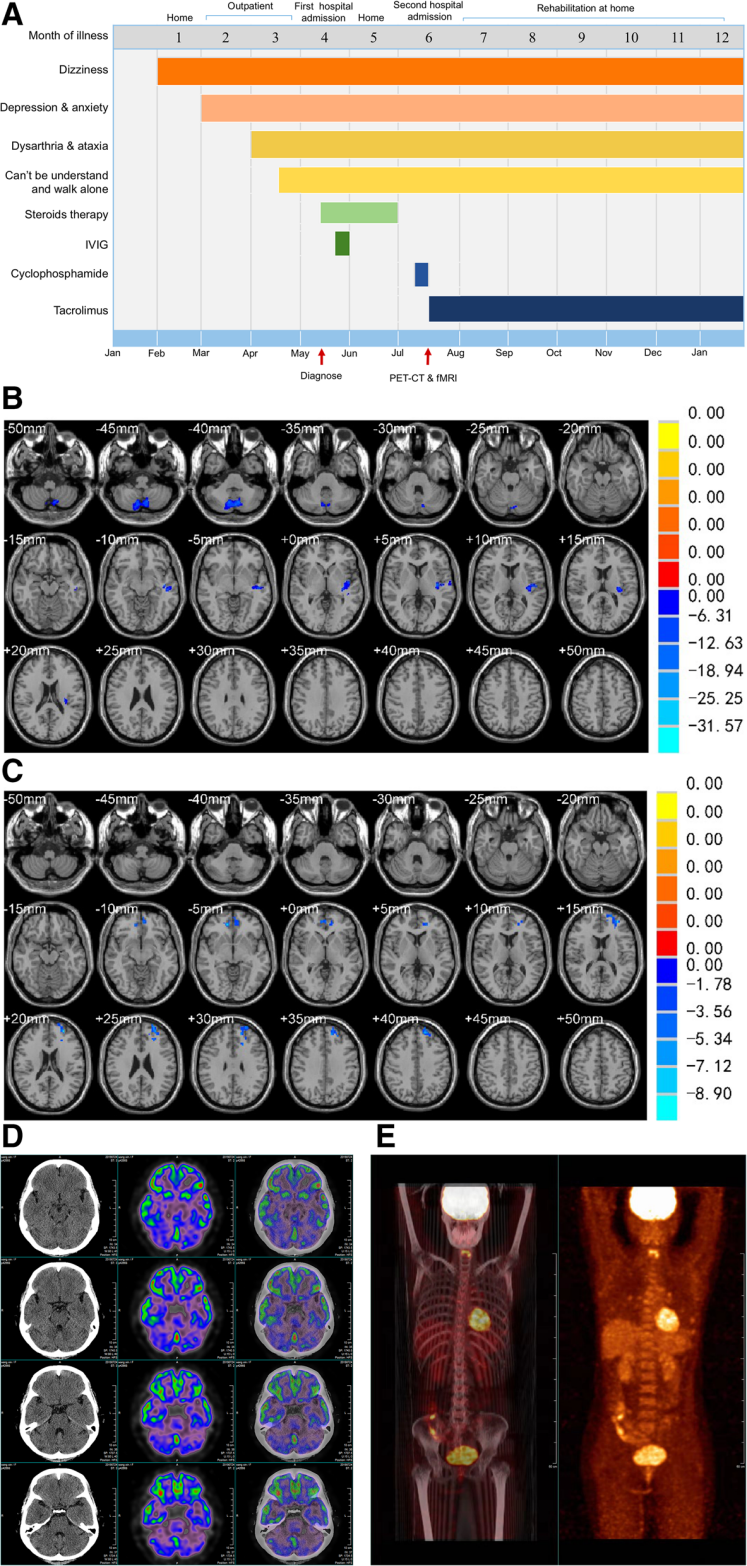
Clinical and imaging features of cerebellar ataxia patient associated with anti-Tr/DNER antibodies. **A**: The symptoms and treatment measures of the patient; **B**: VBM analysis showed reduced cerebellar volume bilaterally, especially in the posterior lobe and uvula of cerebellum, and the middle of the left temporal lobe compared with controls; **C**: The seed-based FC investigation showed lower connectivity between posterior cingulate cortex (PCC)/precuneus and left frontal lobe than that of the control group; **D**: Brain PET-CT showed obvious decreased fluorodeoxyglucose (FGD) uptake in bilateral cerebellum; **E**: whole-body PET-CT showed no malignant changes were found

A 43-year-old woman presented with dizziness for 3 months along with worsening dysarthria and ataxia for 1 month. Apart from severe cerebellar ataxia, she also complained of depression for 2 months, as well as memory loss and blurred vision for 2 weeks. Physical examination showed speech dysarthria and bilateral horizontal gaze-evoked nystagmus that was more obviously towards the right. Finger-nose and heel-shin tests revealed severe ataxia, which had rendered the patient bedridden. Laboratory findings, including complete blood cell count and biochemical, metabolic, infectious, immunologic, and serologic tests, were normal. Cerebrospinal fluid and conventional brain MRI examination were unremarkable. The patient had a Mini Mental Status Examination (MMSE) score of 27 and a Montreal Cognitive Assessment (MOCA) total score of 21. She experienced impairment of short-term memory (2/5), visuospatial functions (1/5), and attention (4/6). The Hamilton Anxiety Scale (HAMA) and Hamilton Depression Scale (HAMA) revealed mild anxiety (15) and moderate depression (23). Anti-Tr antibodies were detected in both her serum (1:10) and cerebrospinal fluid (1:10). Due to the strong association of anti-Tr with malignancy, whole-body contrast computed tomography, ultrasounds of thyroid, breast, and reproductive organs, and bone marrow aspiration were performed for further investigation. However, no malignant changes were found. A diagnosis of anti-Tr positive autoimmune cerebellar ataxia in the absence of malignancy was established and the patient received immune therapy successively. Patient therapy consisted of steroid pulse therapy (5 days of 1 g/d intravenous methylprednisolone sodium succinate, and then 60 mg/d prednisone) followed by intravenous immunoglobulin (0.4 g/kg per day for 5 days). After intravenous therapy, the patient was discharged from the hospital and underwent rehabilitation at home with continual prednisone treatment that was decreased weekly by 5 mg. Patient symptoms and treatment were shown in Fig. 1A.

Upon her re-admission 1 month later, the patient’s major complaint was worsening of the ataxia and an increased cognitive-emotional deficit. In recent years, increasing evidence for a role for the cerebellum in emotion and cognition has emerged [8–12] and the patient was identified as having cerebellar cognitive affective syndrome [13]. The default mode network (DMN) has become the primary and most popular target of resting state networks and is thought to be involved in advanced cognitive functions and emotion [14–16]. Recent fMRI studies have linked activity to cognition and explored how FC is altered in several diseases. The disrupted connectivity within the DMN and remote interregional connectivity related to hubs of DMN have been widely reported in terms of changes related to cognitive deficits. The posterior cingulate cortex (PCC)/precuneus is a pivotal hub in the DMN [17] and also has been linked to many cognitive-emotional disorders [15, 16, 18]. Therefore, to further assess the structural and functional abnormalities and reveal the mechanism underlying the cognitive-emotional deficit in this patient, VBM, PCC and cerebellum seed-based FC analysis were performed at the whole-brain level in addition to PET-CT. Acquisition sequences of sMRI and fMRI were seen in supplementary materials.

VBM analysis showed bilateral reductions in cerebellar volume, especially in the posterior lobe and uvula of cerebellum and the middle of the left temporal lobe in comparison with 6 sex- and age-matched healthy subjects (6 females, 43 ± 2 years; *p *< 0.05; Fig. 1B). Seed-based FC assessment identified reduced connectivity between the PCC/precuneus and left frontal lobe compared to the control group (*p* < 0.05; Fig. 1C). PET-CT revealed bilateral hypometabolism of the cerebellum and relative hypermetabolism of the cerebellar vermis and bilateral frontal lobe (Fig. 1D), but no malignant changes (Fig. 1E). An intravenous of injection of cyclophosphamide was offered at 0.4 g/d for 3 days, followed by Tacrolimus as a long-term immunosuppressive treatment. Unfortunately, the patient failed to improve.

At one-year follow up, the patient repeated the whole-body contrast PET-CT, ultrasounds of thyroid, breasts, and reproductive organs, and bone marrow biopsy with no evidence of malignancy. At this time, her symptoms have not improved.

## Discussion and conclusions

We report a confirmed case of anti-Tr positive autoimmune cerebellar ataxia with no signs of malignancy at 1 year follow up. Studies show that 84.6–94.7 % of patients with this disease also present with Hodgkin’s lymphoma with a median time between diagnosis of cerebellar ataxia and Hodgkin’s lymphoma or any other tumor type being 3.5 months (ranging from 0 to 24 months) [2, 4, 5]. No tumors have been found in our patient, suggesting that, in a few instances, the origin of the anti-Tr antibodies is not a tumor but another unknown cause, such as a para-infectious origin [1]. It is important to note that the follow-up period may have been too short to confidently rule out malignancy for this patient.

Our study illustrates multimodality imaging can be more sensitive than conventional MRI in detection of brain damage and may aid in explaining clinic-radiological discrepancies. In this patient, bilateral reductions in volume and obvious hypometabolism in the cerebellum were observed, which is consistent with her irreversible severe cerebellar ataxia. A postpartum study of patients with anti-Tr/DNER cerebellar ataxia found strong anatomical evidence in the form of the total loss of cerebellar Purkinje cells [4]. Of note, relative hypermetabolism in the cerebellar vermis and bilateral frontal lobe was also observed in our patient, which might be indicative of underlying inflammation [19]. Regions with abnormal hypometabolism and hypermetabolism as identified by PET-CT are also observed in autoimmune encephalitis [19] and paraneoplastic cerebellar degeneration [20]. Reduced middle of the left temporal lobe volume and lower connectivity between the PCC and left frontal lobe can explain patient’s cognitive-emotional deficit. These structures are all parts of the DMN, which has been linked to cognitive-emotional processing [17]. Grimm and Simone et al. investigated neural activity in the DMN during different emotional tasks in healthy subjects and patients with major depressive disorder in an event-related fMRI design. They found decreased negative BOLD responses in the PCC and ventromedial prefrontal cortex in patients with major depressive disorder that correlated with depression severity and feelings of hopelessness [15]. The PCC and ventromedial prefrontal cortex have also been shown to be involved in short-term memory, visuospatial functions, and attention using a number of fMRI studies [14, 18, 21]. Involvement of extra-cerebellar regions is also supported by neuroanatomy studies. In rats, anti-Tr antibody immunoreactivity was not confined to the Purkinje cells of the cerebellum but was also observed in the plexiform layers of the hippocampus and neocortex [22, 23].

The cerebellum may also play a role in cognitive-emotional deficits in patient. Anatomically, there are extensive anatomical connections between the cerebellum and associated paralimbic areas, such as the temporoparietal junction, lateral temporal cortex, PCC, inferior frontal gyrus, amygdala, and insula [24, 25]. Most of these are part of the DMN described above and involved in processing of cognition and emotions. Additional insight was derived from VBM studies [9, 26]. For example, Olivito et al. assessed the relationship between cerebellar gray matter (GM) atrophy and neuropsychological scores in patients with spinocerebellar ataxia 2 using VBM and found GM loss in the cognitive posterior lobules (VI, Crus I, Crus II, VIIB, and IX) that correlated with visuospatial, verbal memory, and executive tasks [26]. Further evidence has been provided by RS-fMRI studies that showed altered FC between cerebellar and cerebrum regions, which are known to be related to cognition and emotion, in patients with spinocerebellar ataxia 2, depression, and Alzheimer’s disease [9, 27–30]. In our patient, VBM analysis revealed atrophy in both the DMN structure and cerebellum. FC analysis demonstrated decreased connectivity in the DMN. Based on the studies mentioned above, we suggest the cognitive-emotional deficits in our patient were caused by disfunction of the cerebellum and DMN.

Notably, the reduced left frontal lobe-precuneus connectivity seen in our patient is nonspecific. Recent work has shown impairment of motor and cognitive cerebellar-neocortical networks in chronic ataxias, such as spinocerebellar ataxia 17 [31]. Similarly, other groups have recently outlined deficits in cerebellar-neocortical networks and cerebellar-striatal connectivity in patients with multiple system atrophy (MSA) [32] and overactivity in the olivary-cerebellar-thalamic network in patients with an essential tremor [33]. Here, we give the first insight into cerebellar-neocortical network functionality in anti-Tr positive autoimmune cerebellar ataxia. However, caution is needed when interpreting our results as this study consisted of only one patient. Our findings should be evaluated using larger cohort studies.

In conclusion, we report a confirmed case of anti-Tr positive autoimmune cerebellar ataxia with no tumor signs at 1 year follow up. A combination of structural MRI, functional MRI, and brain PET-CT has diagnostic and prognostic value in patients with suspected anti-Tr/DNER encephalitis. Disfunction of the cerebellum and DMN may contribute to the cognitive-emotional deficits seen in this disorder.

## Supplementary Information

**Additional file 1.**

## Data Availability

All data used in this study are available from the corresponding author on reasonable request.

## Abbreviations

DNER: Delta/Notch-like epidermal growth factor-related receptor
MRI: Magnetic resonance imaging
VBM: Voxel-based morphometry
RS-fMRI: Resting state functional magnetic resonance imaging
BOLD: Blood oxygen level-dependent
FC: Functional connectivity
PET-CT: Positron emission tomography-computed tomography
CSF: Cerebrospinal fluid
MMSE: Mini Mental Status Examination
MOCA: Montreal Cognitive Assessment
HAMA: Hamilton Anxiety Scale
HAMD: Hamilton Depression Scale
DMN: Default mode network
PCC: Posterior cingulate cortex
GM: Gray matter
MSA: Multiple system atrophy
