# Supplementary material for: Combination of structural MRI, functional MRI and brain PET-CT provide more diagnostic and prognostic value in patients of cerebellar ataxia associated with anti-Tr/DNER: a case report
Source: BMC Neurol. 2021 Sep 24;21:368. doi: 10.1186/s12883-021-02403-5 (PMC8461997; doi:10.1186/s12883-021-02403-5)
Supplement: Supplementary file 1 — Additional file 1. [file 12883_2021_2403_MOESM1_ESM.docx]

MR images were acquired using a 3.0 T MRI scanner (Siemens Trio) with a standard 8-channel head coil. Foam padding and ear plugs was used to minimize the head motion and make subjects comfortable. The included subjects were informed to relax, close eyes, and not to sleep during scanning. High-resolution 3D-T1 (TR = 8.3 ms, TE = 3.3 ms, flip angle = 15°, thickness/gap = 1.0/0 mm, FOV = 240 × 240 mm, matrix = 256 × 192) and T2-FLAIR-weighted images (TR = 8000 ms, TE = 126 ms, TI = 1500 ms, thickness/gap = 5.0/1.5 mm, FOV = 240 × 240 mm, matrix = 256 × 192) were also acquired. RS-fMRI data were acquired using an echo-planar image (EPI) pulse sequence (TR: 2000 ms, echo time: 30 ms, flip angle: 90°, slice thickness: 5 mm, no slice gap, field of view: 240 × 240 mm2, voxel size: 3.75 × 3.75 × 5 mm3, 30 axial slices, 200 volumes). VBM was calculated by VBM8 toolbox (http://dbm.neuro.uni-jena.de/vbm.html) and SPM8 (https://www.fil.ion.ucl.ac.uk/spm/) software. First, the structural images were segmented into GM, WM and cerebrospinal fluid. Then the structural images were normalized to Montreal Neurologic Institute (MNI) space using VBM8. Finally, Grey matter volume images were smoothed with a Gaussian kernel of 8 mm FWHM. Functional image preprocessing was carried out using the SPM12 software (https://www.fil.ion.ucl.ac.uk/spm/software/spm12) and DPRSFA V4.4 (http://rfmri.org/DPARSF). The following steps were performed:(1) remove first 10 timepoints; (2) slice timing correction; (3) head motion correction. The datasets with head motion exceeding 3 mm in translation and 3° in rotation were excluded; (4) spatial normalization, the individual T1 image was co-registered to the mean functional image and the co-registered T1 image was segmented into gray matter (GM), white matter (WM) and cerebrospinal fluid (CSF) using unified segmentation algorithm. Then the co-registered functional images were normalized into Montreal Neurologic Institute (MNI) space and resampled to 3×3×3 mm³ resolution; (5) detrend; (6) covariates regressed including the Friston-24 head motion parameters, the WM signal, the cerebrospinal fluid signal and adding the mean of the time series back; (7) band-pass filter (0.01-0.08Hz) was applied after calculating Functional Connectivity; (8) all post-processing images were smoothed with a Gaussian kernel of 4 mm FWHM.
